# National Tuberculosis Genotyping and Surveillance Network: Analysis of the Genotype Database

**DOI:** 10.3201/eid0811.020313

**Published:** 2002-11

**Authors:** Lauren S. Cowan, Jack T. Crawford

**Affiliations:** * Centers for Disease Control and Prevention, Atlanta, Georgia, USA

**Keywords:** *Mycobacterium tuberculosis*, strain typing

## Abstract

As part of the National Tuberculosis and Genotyping Surveillance Network, isolates obtained from all new cases of tuberculosis occurring in seven geographically separate surveillance sites from 1996 through 2000 were genotyped. A total of 10,883 isolates were fingerprinted by the IS6110-restriction fragment length polymorphism method, yielding 6,128 distinct patterns. Low-copy isolates (those with six or fewer bands) were also spoligotyped. The distribution of specific genotype clusters was examined. Databases were also examined for families of related genotypes. Analysis of IS6110 patterns showed 497 patterns related to the W-Beijing family; these pattens represent 946 (9%) of all isolates in the study. Six new sets of related fingerprint patterns were also proposed for isolates containing 6–15 copies of IS6110. These fingerprint sets contain up to 251 patterns and 414 isolates; together, they contain 21% of isolates in this copy number range. These sets of fingerprints may represent endemic strains distributed across the United States.

The National Tuberculosis Genotyping and Surveillance Network was created by the Centers for Disease Control and Prevention (CDC) to determine the relative frequency of Mycobacterium tuberculosis strains in specific geographic areas, the extent of spread of related strains in communities, and the impact of IS6110 fingerprinting on tuberculosis (TB) control. From 1996 through 2000, the TB genotyping network laboratories fingerprinted 10,883 isolates (one isolate per newly diagnosed case of TB) from seven sentinel surveillance sites in the United States: the states of Arkansas, Maryland, Massachusetts, Michigan, and New Jersey, along with four counties in Texas and six counties in California. Key components of the project included the establishment of standard methods and use of specialized software, the BioImage Whole Band Analyzer version 3.4 (BioImage, Ann Arbor, MI), for pattern analysis. The following were created as part of this study: databases containing the images of IS6110 patterns for all isolates at each sentinel surveillance site; a network database that includes all distinct spoligotype patterns; and an epidemiologic database (Epi-Info) for information about sentinel surveillance site, case report date, IS6110 pattern designation, and secondary typing results for each patient. The final network database of fingerprints contains 6,128 patterns.

## High-Copy Isolates

We report here an overview of the contents of the TB genotyping network fingerprint database, including the distribution of isolates at sentinel surveillance sites, genotype patterns that occurred with high frequency, the extent of previously described genotype families, and new families of related fingerprints. This analysis should not be considered exhaustive but rather a summary of our observations and an introduction to the types of data that can be derived.

## Methods

Methods for IS6110 fingerprinting, spoligotyping, and compiling the network databases are described elsewhere (1). The distribution of isolates by sentinel surveillance site, fingerprint pattern, and spoligotype (for isolates with six or fewer copies of IS6110) was derived from the Epi Info database. The spoligotype patterns are reported in octal code by the convention previously described (2). IS6110 fingerprint patterns start with the prefix FP and spoligotype patterns with SP.

We used the BioImage Whole Band Analyzer software package version 3.4 to analyze fingerprint patterns in the genotyping network fingerprint database. The bands in two patterns were compared at two levels. First, bands in two patterns were identified as matched bands if the size of the bands differed by <2.5%. Next, the interband spacing between matched bands in the two patterns was compared; a limit of 95% for variation in interband spacing was used. The Jaccard coefficient of similarity between two patterns, A and B, was used to calculate the percentage match between two patterns: 100 x number of matched bands /(number bands in A + number bands in B - number of matched bands).

## Results and Discussion

**Figure 1:**
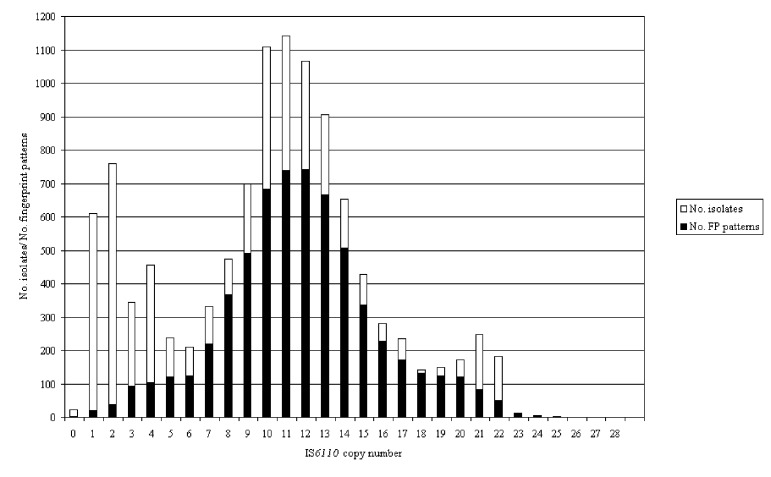
Distribution of all isolates and fingerprint patterns by number of copies of IS*6110*. The light bars show the distribution of isolates; the dark bars show the distribution of fingerprint patterns.

Isolates from 10,883 patients from seven sentinel surveillance sites were fingerprinted by using the IS6110 restriction fragment length polymorphism (RFLP) method: Arkansas, 709; California, 2,514; Massachusetts, 986; Maryland, 1,180; Michigan, 1,471; New Jersey, 2,113; and Texas, 1,910. From these isolates, 6,128 distinct fingerprint patterns were identified and included in the final genotyping network database. Analysis of the IS6110 copy number of the isolates confirmed the previously described bimodal distribution (Figure 1) (3). This distribution has been used to separate isolates of M. tuberculosis into two groups: isolates with six or fewer copies of IS6110 are classified as low-copy isolates and those with more than six copies as high-copy isolates. The greatest numbers of patterns occurred in the 9–14 copy-number range (Figure 1). Clustering of isolates on the basis of matching fingerprint patterns is summarized in Table 1. Clustering was very high among the low-copy isolates, which supports the requirement for secondary typing of these isolates. Clustering decreased with increasing copy number; copy numbers 21 and 22, which included large outbreaks, were the exceptions.

**Table 1:** Distribution of isolates and fingerprint patterns by number of copies of IS6110

IS*6110* copy no.	No. patterns	No. isolates	No. clusters^a^	No. clustered isolates (%)	Average cluster size (range)
0	1	22	1	22 (100)	22 (22)
1	17	610	9	602 (99)	67 (2–291)
2	36	759	16	739 (97)	46 (2–456)
3	92	345	40	293 (85)	7.3 (2–49)
4	102	456	28	382 (84)	14 (2–212)
5	119	237	37	155 (65)	4.2 (2–13)
6	121	209	21	109 (52)	5.2 (2–20)
7	217	332	40	155 (47)	3.9 (2–29)
8	364	474	56	166 (35)	3.0 (2–12)
9	489	699	78	288 (41)	3.7 (2–20)
10	681	1,108	112	539 (49)	4.8 (2–105)
11	737	1,143	116	522 (46)	4.5 (2–70)
12	738	1,067	114	443 (42)	3.9 (2–27)
13	663	906	83	326 (36)	3.9 (2–23)
14	505	653	51	199 (30)	3.9 (2–27)
15	333	428	35	130 (30)	3.7 (2–15)
16	225	282	17	74 (26)	4.4 (2–21)
17	169	236	16	83 (35)	5.2 (2–46)
18	128	143	9	24 (17)	2.7 (2–5)
19	121	149	17	45 (30)	2.6 (2–7)
20	118	172	23	77 (45)	3.3 (2–14)
21	81	248	15	182 (73)	12 (2–100)
22	48	182	12	146 (80)	12 (2–102)
23	13	13	0		
24	5	5	0		
25	2	2	0		
26	1	1	0		
27	1	1	0		
28	1	1	0		
All	6,128	10,883	946	5,701 (52)	6.0 (2–456)

^a^Number of fingerprint patterns reported for more than one isolate.

**Figure 2:**
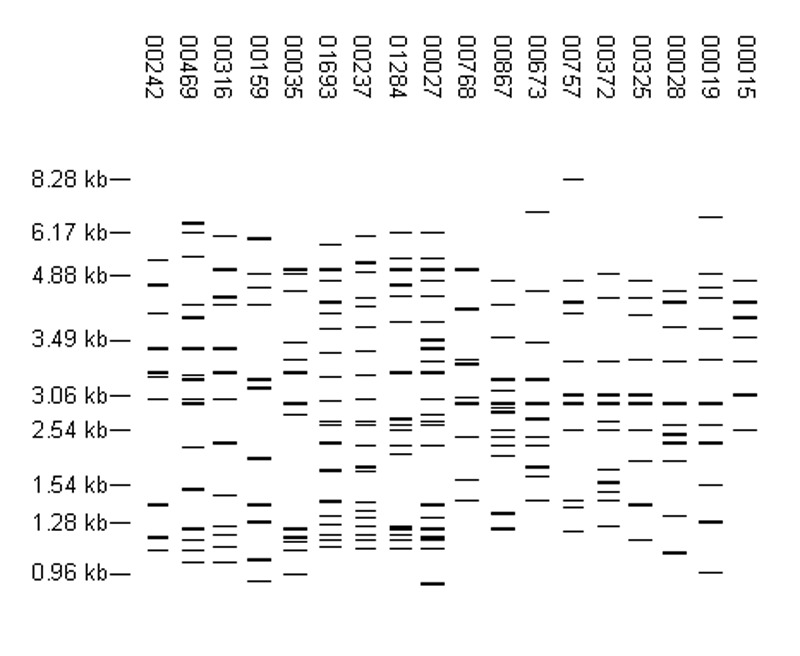
High-frequency, high-copy fingerprint patterns. Each pattern was reported for ≥20 isolates. The distribution of isolates with these patterns by sentinel surveillance site is shown in Table 2.

The 8,245 isolates with more than six copies of IS6110 yielded 5,640 fingerprint patterns. Of these patterns, 4,846 (85.9%) were identified for a single isolate, and 3,399 isolates were grouped into 794 fingerprint-defined clusters. Of these clusters, 557 contained isolates from a single site. The clusters contained up to 105 isolates, but 683 (86.0%) of the clusters contained only two to five isolates. In fact, only 18 clusters contained 20 or more isolates. The distribution of isolates in these 18 clusters is shown in Table 2, and the fingerprint patterns are shown in Figure 2. For 11 of the 18 largest clusters, >90% of the isolates in the cluster were from a single site.

**Table 2:** Distribution of isolates with high-copy fingerprint patterns reported with high frequency^a, b^

			No. isolates/site						
FP	No. bands	No. isolates	AR	CA	MA	MD	MI	NJ	TX
00015	7	29	0	0	0	2	0	27	0
00019	12	27	4	7	0	2	3	2	9
00027	22	102	0	0	0	0	102	0	0
00028	11	70	0	0	0	0	70	0	0
00035	13	33	0	0	0	0	0	0	33
00159	11	24	0	0	0	0	0	0	24
00237	21	100	12	0	0	2	0	1	85
00242	10	105	6	1	1	0	1	0	96
00316	14	27	3	22	2	0	0	0	0
00325	11	20	15	1	0	0	0	4	0
00372	12	20	11	0	0	0	0	0	9
00469	16	21	1	0	0	0	0	0	20
00673	11	25	0	19	0	0	0	4	2
00757	11	20	0	0	0	17	0	3	0
00768	9	20	0	0	0	0	0	20	0
00867	14	24	0	24	0	0	0	0	0
01284	17	46	0	0	0	0	0	0	46
01693	21	40	0	0	1	0	0	39	0

^a^FP, fingerprint; AR, Arkansas, CA, California; MA, Massachusetts; MD, Maryland; MI, Michigan; NJ, New Jersey, TX, Texas. ^b^Patterns reported for ≥20 isolates.

**Figure 3:**
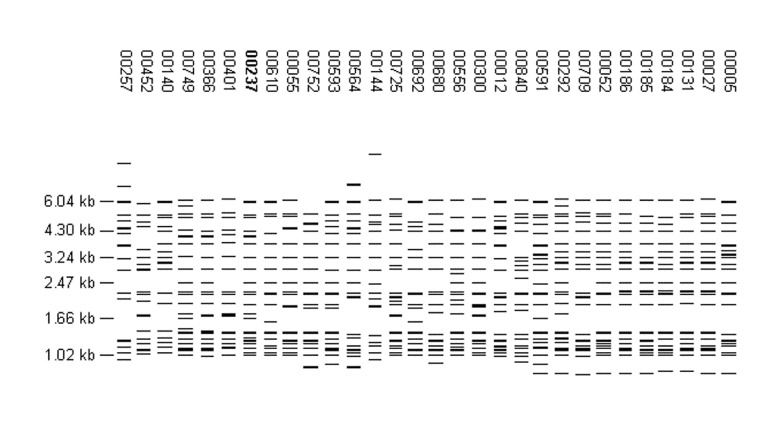
Examples of fingerprint patterns in the W-Beijing family that were visually identified as being similar to the prototype pattern, FP 00237.

One of the largest clusters (FP 00237, 100 isolates) corresponds to M. tuberculosis strain 210, a member of the W-Beijing family that was shown in previous studies to be disseminated across the United States (3). In the network, FP 00237 was associated with large clusters in Arkansas and Texas and was also reported by Maryland and New Jersey. Two additional patterns associated with large clusters, FP 00027 (102 isolates in Michigan) and FP 01284 (46 isolates in Texas), were similar to FP 00237. The Beijing family of strains has received considerable attention because of its association with several large outbreaks, frequent association with multidrug resistance, and emergence in selected populations, particularly in the former Soviet Union (4,5). All Beijing isolates share a characteristic spoligotype (000000000003771); however, in this study, spoligotyping was not performed for high-copy isolates. Other molecular criteria that define W-Beijing strains include insertions of IS6110 in the dnaA-dnaN region (A1 insertion) and in the NTF region and an empirical fingerprint pattern that contains 15 to 24 bands and is similar to that of strain W (4). To estimate the occurrence of Beijing isolates in our study, all patterns with 16 to 24 bands were visually compared to FP 00237. The W fingerprint was easily identified among the patterns with 17 or more bands; however, we were less confident about identifying it in those with 16 bands and did not include them in this analysis. Of the 688 patterns analyzed, 497 (72.2%) were similar to FP 00237. Examples can be seen in Figure 3. Nearly all of the individual patterns, 480 (97%), were reported by a single site. These 497 patterns represent 946 isolates, 82% of all isolates with >17 copies of IS6110 and 9% of all isolates in the study (Arkansas, 3%; Maryland, 4%; New Jersey, 7%; Massachusetts, 9%; and California, Michigan, and Texas, 11%). The distribution of these isolates by site is reported in Table 3.

**Table 3:** Distribution of isolates in genotype families^a^

			No. isolates/site								
	No. patterns	No. isolates	AR	CA	MA	MD	MI	NJ	TX	Clustered isolates (%)	Average no. copies of IS*6110*/isolate (range)
FP sets											
W-Beijing	497	946	22	279	88	49	162	144	202	56	19.9 (17–27)
Set A	141	190	37	18	14	19	43	29	30	39	13.1 (10–15)
Set B	97	162	14	33	20	4	15	33	43	52	11.0 (7–15)
Set C	251	414	4	295	9	15	20	53	18	52	11.9 (8–15)
Set D	181	275	11	37	44	32	27	102	22	48	8.9 (6–13)
Set E	119	137	0	37	5	16	16	56	7	20	12.9 (9–15)
Set F	177	321	24	44	31	47	47	71	57	55	9.4 (6–14)
FP 17	54	411	31	59	31	64	65	72	89	70	4.5 (3–6)
											
Spoligotype family											
EA-I	161	558	7	247	71	46	62	51	74	56	1.8 (1–6)
X	113	1,291	61	267	73	98	232	270	290	83	3.1 (1–6)
Haarlem	11	47	1	1	5	23	2	5	10	45	4.9 (1–6)
LAM-1	5	6	0	1	2	2	0	1	0	33	3.7 (1–6)
LAM-2	4	54	0	0	0	51	0	2	1	81	2.6 (1–6)
bovis	19	32	2	8	1	2	5	8	6	41	1.4 (1–5)
africanum	10	19	0	1	1	11	2	3	1	47	4.4 (3–6)

^a^ FP, fingerprint, AR, Arkansas, CA, California; MA, Massachusetts; MD, Maryland; MI, Michigan; NJ, New Jersey, TX, Texas; EA-I, East African-India^;^ LAM, Latin American-Mediterranean.

**Figure 4:**
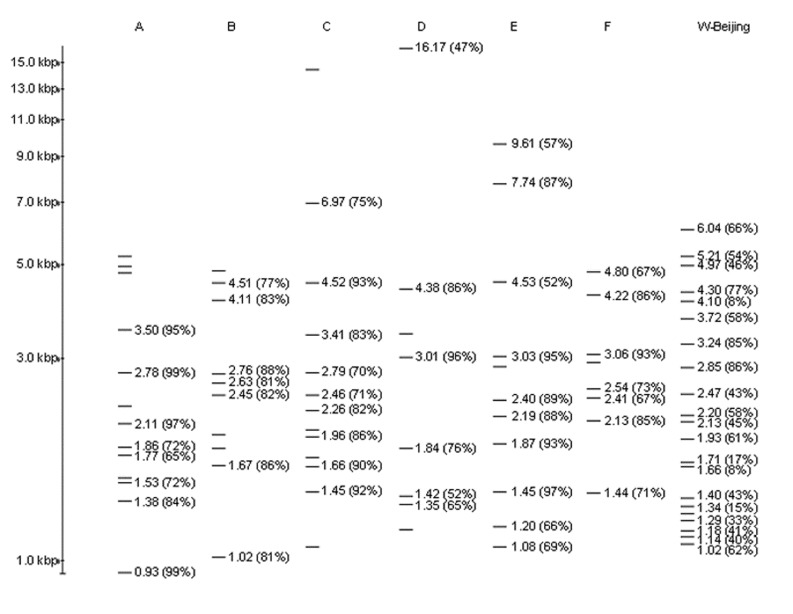
Prototype patterns for genotype sets. Set A: FP 00102; Set B: FP 04924; Set C: FP 02789; Set D: FP 02646; Set E: FP 02170; Set F: FP 04666; and W-Beijing family: FP 00237. The size of each band and the percentage of patterns in the set with each band are indicated on the pattern obtained by restriction fragment length polymorphism typing. For sets A–, only the bands common to the six prototype patterns were analyzed.

Because only one of the molecular criteria (overall fingerprint pattern) could be applied, isolates with these patterns cannot be definitively called W-Beijing. All of the insertion sites in strain 210, FP 00237, have been defined by sequencing (6). To identify conserved insertion sites, we determined the percentage of the 497 patterns that contained each of the bands in FP 00237 (Figure 4). Nine bands were found in >50% of the patterns, and two were present in >85%. A common feature of these fingerprint patterns is a group of smaller bands (1.0 to 1.5 kb) that are difficult to resolve. Variation in band identification resulted in some of the heterogeneity of the patterns in the database. W-Beijing strains likely account for a large portion of Beijing isolates, but other Beijing strains exist. FP 00242 (reported for 96 isolates in Texas; fingerprint pattern shown in Figure 2) shares only a few bands with FP 00237, but isolates with this pattern have the Beijing spoligotype (Teresa Quitugua, pers. comm.).

**Figure 5:**
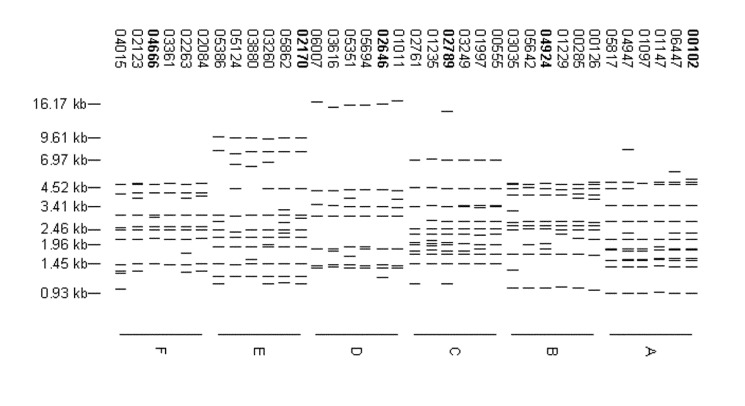
Prototype fingerprint patterns used to define genotype sets A–F. The prototype patterns were identified by first matching all patterns with 6–15 bands to each other at a matching threshold of 50%. The pattern that matched the greatest number of other patterns and the five patterns that matched this pattern and the greatest number of other patterns were selected as the prototype patterns for a set. All patterns that matched one of the six prototype patterns were then assigned to this set. The process was repeated by using the remaining unassigned patterns to create the six sets of fingerprint patterns. For each set, the six prototype patterns were visually examined for common bands. The mean, standard deviation, and coefficient of variation of each band in the six patterns were calculated, and bands with a coefficient of variation >2% were eliminated. The mean band size of the selected bands was used to identify which of the six patterns contained the common bands closest to the average band size (pattern identified with bold font).

To identify other large families in the database, we analyzed all patterns having 6 to 15 bands (4,846 patterns). Since the BioImage software cannot create a dendrogram for more than 1,250 patterns, patterns were compared to each other by using an arbitrarily chosen matching threshold of 50% to identify those that matched a large number of other patterns. For a 50% match, two thirds of the bands in two patterns with equal band number must match. Six sets of related fingerprints, designated A through F, were defined; each consisted of six prototype patterns (Figure 5) along with all of the patterns that matched the prototypes. Data on these sets are summarized in Table 3. The isolates in each set appear widely dispersed across the sites, and the patterns likely represent endemic strains in the United States. Key bands in each set were determined in comparison to the common bands in the prototype patterns as described above for FP 00237 (Figure 4). Sequencing the IS6110 insertion sites corresponding to these key bands would allow isolates belonging to these sets to be rapidly identified with microarray techniques or the reverse dot-blot (“insite”) assay we have described previously (7).

The sets described here are certainly not the only sets of related patterns in the database, nor are they necessarily novel. The patterns in set A are similar to the patterns for M. tuberculosis strains H37Rv and H37Ra (8); the eight common bands in the prototype patterns for set A are also found in the patterns for these two laboratory strains. The patterns in set D appear similar to those of the Haarlem family (9). Interestingly, 1,404 (29.0%) of the patterns with 6 to 15 bands did not match any other pattern at the 50% matching threshold, suggesting a substantial number of orphan strains in this study.

### Low-Copy Isolates

**Figure 6:**
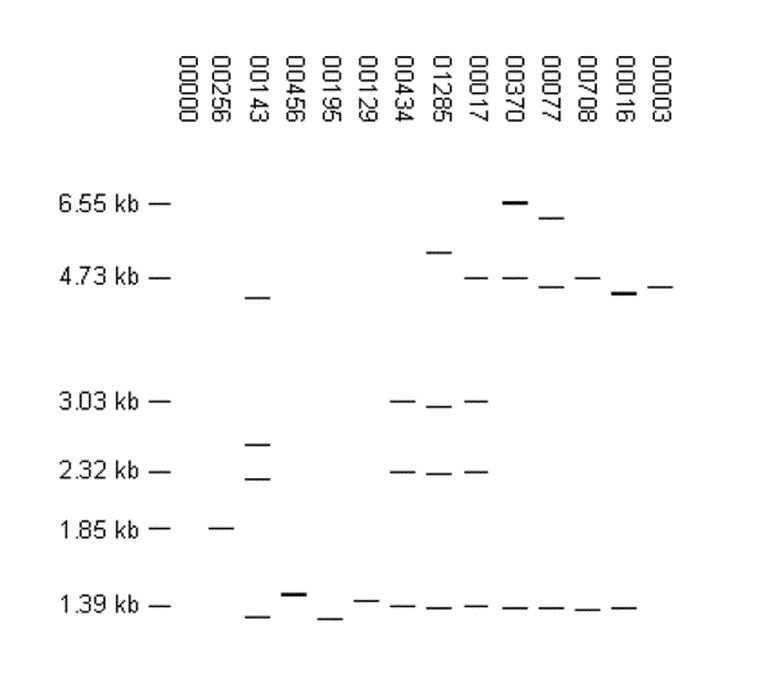
High-frequency, low-copy fingerprint patterns. Each pattern was reported for ≥20 isolates. The distribution of isolates with these patterns by sentinel surveillance site is shown in Table 4.

Of the 457 fingerprint patterns identified among the 2,507 low-copy isolates, 314 (68.7%) were reported for a single isolate, and 143 patterns grouped 2,193 isolates into clusters. Clustering was much higher in low-copy isolates (87.5%) than in high-copy isolates (41.2%). Most isolates were in a few large clusters; 14 clusters contained 1,601 (63.9%) low-copy isolates. The distribution of isolates in the largest clusters across the sentinel surveillance sites is shown in Table 4, and the fingerprint patterns are shown in Figure 6.

**Table 4:** Distribution of isolates with low-copy fingerprint patterns reported with high frequency^a,b^

			No. isolates/site^a^							
FP	No. bands	No. isolates	AR	CA	MA	MD	MI	NJ	TX	No. spoligotypes^c^
00000	0	21	0	11	0	7	1	0	2	11
00003	1	87	2	32	13	12	11	12	5	26
00016	2	429	47	0	22	67	116	56	121	70
00017	4	201	12	15	13	39	29	48	45	45
00077	3	49	0	0	7	12	16	0	14	14
00129	1	289	1	84	60	35	29	66	14	92
00143	4	28	2	1	0	9	5	10	1	5
00195	1	148	5	76	0	5	13	3	46	52
00256	1	28	2	7	1	2	4	7	5	16
00370	3	38	0	9	0	0	0	26	3	9
00434	3	21	1	4	1	0	10	2	3	9
00456	1	32	0	0	0	0	0	32	0	18
00708	2	207	0	184	0	0	3	20	0	19
01285	4	23	0	0	0	0	0	1	22	4

^a^FP, fingerprint, AR, Arkansas; CA, California; MA, Massachusetts; MD, Maryland; MI, Michigan; NJ, New Jersey, TX, Texas. ^b^Patterns reported for ≥20 isolates. ^c^Number of different spoligotypes reported for isolates with this pattern.

Spoligotype results were available for most low-copy isolates (2,507 of 2,638 isolates). Isolates collected in Arkansas before 1998 were not spoligotyped (97 of 210 isolates) nor were most isolates collected in Maryland before 1998 that had unique fingerprint patterns (23 of 323 isolates). Of the 495 spoligotypes identified among the low-copy isolates, 322 were reported for a single isolate, and 173 grouped 2,185 isolates into clusters. In this study, the clustering of low-copy isolates by spoligotyping (87.2%) was only slightly lower than clustering by fingerprinting (87.5%). Analysis of the isolates by IS6110 copy number showed that spoligotyping performed better than fingerprinting only for those isolates with fewer than four copies of IS6110 (data not shown). Similar to the results obtained with fingerprinting, most isolates are in large clusters; 1,481 isolates are in the 20 largest clusters. The spoligotypes for these clusters as well as the distribution of these isolates by site and IS6110 copy number are listed in Table 5.

**Table 5:** Distribution of isolates with spoligotypes reported with high frequency^a,b^

			No. isolates/site							No. isolates/IS*6110* copy number						
SP	Octal code^c^	No. isolates	AR	CA	MA	MD	MI	NJ	TX	1	2	3	4	5	6	No. fingerprint patterns^d^
2	777777777760771	84	8	25	10	2	8	14	17	40	11	5	15	3	10	30
3	777776777760771	331	16	57	16	3	40	48	151	5	82	25	122	61	36	77
9	777776777760601	288	20	35	18	3	89	51	72	3	190	72	17	6	0	44
15	777777777413771	50	0	19	11	2	5	3	10	26	6	3	7	4	4	21
16	777777777416761	21	0	0	16	0	0	2	3	10	2	0	2	6	1	10
19	777777774413771	99	0	69	18	0	7	4	1	83	5	6	3	1	1	17
27	701776777760601	131	0	130	1	0	0	0	0	0	129	1	0	1	0	4
28	700036777760771	34	0	4	7	1	2	18	2	1	6	19	0	8	0	12
29	700076777760771	46	2	7	5	1	7	13	11	0	0	0	27	11	8	13
30	700036777760731	44	0	3	3	0	10	16	12	0	2	42	0	0	0	4
72	700076777760671	38	4	2	2	0	13	14	3	0	0	0	28	9	1	7
75	777776407760601	57	0	1	0	0	0	55	1	41	14	2	0	0	0	4
91	477777777741071	24	0	0	1	0	0	23	0	18	3	0	0	1	2	8
300	777756777760601	41	0	0	1	38	2	0	0	0	4	9	14	0	14	9
540	477777777413071	44	1	17	5	9	10	1	1	25	4	5	3	1	6	21
545	037776777760601	31	0	0	0	0	30	0	1	0	30	1	0	0	0	2
546	777777777413731	26	0	17	0	3	4	0	2	20	3	0	2	0	1	7
560	777777777760601	20	8	0	0	0	0	0	12	0	17	2	1	0	0	4
562	777777776413771	21	4	0	0	0	0	0	17	18	2	1	0	0	0	4
900	776377777740731	51	0	0	0	51	0	0	0	0	36	11	2	0	2	11

^a^AR, Arkansas, CA, California; MA, Massachusetts; MD, Maryland; MI, Michigan; NJ, New Jersey, TX, Texas. ^b^Spoligotypes reported for ≥ 20 isolates. ^c^The 43-digit spoligotype pattern is reported in the standard octal code format (2). ^d^Number of different fingerprint patterns reported for isolates with this spoligotype.

Neither fingerprinting nor spoligotyping provided great discriminatory power among low-copy isolates, but the combination of the two methods gave slightly better results. The number of spoligotypes identified per fingerprint pattern ranged from 1–92 spoligotypes, and the number of fingerprint patterns identified per spoligotype ranged from 1–77 patterns. Combining the fingerprinting and spoligotyping data resulted in the identification of 987 distinct genotypes; 745 genotypes were unique, and 242 grouped 1,762 isolates into clusters. These genotype clusters contained up to 167 isolates. Performing the secondary typing method decreased the number of clustered isolates by nearly 20%, but clustering was still much higher among the low-copy isolates (70.2%) than among the high-copy isolates (41.2%).

In our recent study of low-copy isolates from Michigan, we noted numerous patterns with similarities to FP 00017 (10). In this study, 201 isolates from all seven sites had FP 00017. When the three lower bands (1.39, 2.32, and 3.03 kb) in FP 00017 were matched to all patterns having three to six bands, 54 patterns representing 411 isolates were identified. The distribution of these isolates by site can be seen in Table 3. Of note, M. tuberculosis strain CDC1551 has FP 00017 (11), but none of the study isolates with this fingerprint had the spoligotype corresponding to strain CDC1551 (700076757760771).

**Figure 7:**
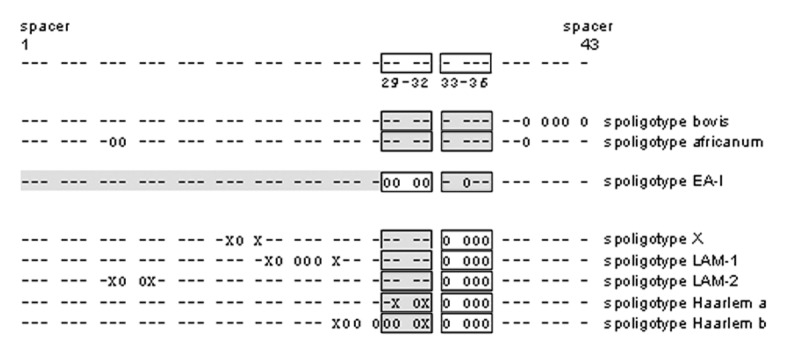
Motifs used to assign spoligotype patterns to spoligotype families. Each spoligotype was analyzed for the bovis (16), africanum (17), East-African-Indian (EA-I) (13,18), X (13), Latin American-Mediterranean 1 and 2 (13,18), and Haarlem a and b spoligotype motifs (10). Each motif definition was modified from the original references to ensure that motifs were not identified in a spoligotype pattern due to an unrelated deletion at the spacers of interest; each of the motif-defining absent spacers must be flanked on both sides by the adjacent spacer. The 43 spacers in the spoligotype pattern are classified with symbols: X: spacer must be present; 0: spacer must be absent; -: spacer may or may not be present; spacers in shaded boxes: at least one of the spacers in the box must be present.

Spoligotypes have been divided into clades or families on the basis of commonly observed motifs (Figure 7) (12). First, spoligotypes can be subdivided on the basis of spacers 33–36. Only M. tuberculosis complex genotypic group 1 strains (M. bovis, M. africanum, and some M. tuberculosis strains) have spacers 33–36 (13,14). Four spoligotype motifs have been identified among group 1 isolates: bovis (15), africanum (16), Beijing (5), and East African-Indian (EA-I) (12,17) (Figure 7). The remaining spoligotypes that lack spacers 33–36 can be subdivided into two subgroups on the basis of spacers 29–32. Isolates with at least one of spacers 29–32 are likely to be isolates in M. tuberculosis genotypic groups 2 or 3. Isolates without spacers 29–32 have a deletion in the direct repeat locus that is too large to definitively assign to a genotypic group. Four specific motifs have been identified among the spoligotypes associated with non–genotypic group 1 isolates: Haarlem (9), Latin American and Mediterranean 1 and 2 (12,17), and X (12) (Figure 7). Of the 495 spoligotypes observed for low-copy isolates, 323 contained one of the eight defined motifs. This allowed 2,007 (80.1%) low-copy isolates to be assigned to a spoligotype family; the data for each family are summarized in Table 3. The majority (51.5%) of the low-copy isolates belonged to family X. The only published information regarding this motif indicated that it is highly prevalent in some English-speaking countries (12). In our study, 1,036 (70.3%) of isolates with two to four copies of IS6110 belonged to family X. The second largest spoligotype family was family EA-I. Isolates with this motif belong to group 1 (13) and have up to nine copies of IS6110 (17). Our isolates that belonged to this family had one to six copies of IS6110, but 378 (67.7%) possessed a single copy. In fact, 62.7% of isolates with a single copy of IS6110 belonged to the EA-I family. The remaining spoligotype families grouped only a few isolates, probably because isolates in these families are mostly high copy (9,17), and this occurrence should not suggest that these spoligotype families are uncommon in the United States. Thirty-two isolates were classified as M. bovis and 19 as M. africanum, solely by spoligotype motifs; no additional tests were conducted to confirm this classification.

After isolates were assigned to a spoligotype family, fingerprint clusters of isolates were examined for consistency with the spoligotype family assignment. We were surprised to identify several fingerprint patterns that have isolates with very different spoligotype patterns. For example, FP 00017 (Figure 6) and FP 00104 (a five-band pattern) share four bands in common with a size difference of <1% and also have two spoligopatterns in common. SP 3 (777776777760771) is a very common pattern among M. tuberculosis group 2 and 3 isolates (13), whereas SP 290 (330777777767671) has a motif associated with M. africanum isolates (group 1). The spoligotype patterns are clearly divergent, indicating either that the strains independently acquired three copies of IS6110 at the same insertion sites or that they have different IS6110 insertions that coincidentally yield PvuII fragments of the same length.

Most of the other examples of isolates clustered by IS6110 with divergent spoligotypes are among isolates with one or two copies of IS6110. Mathema et al. (18) investigated differences among 66 isolates with FP 00129 (one band of 1.40 kb); 26 had group 1 spoligotypes, and 40 had group 2 or 3 spoligotypes. In most isolates with a single copy of IS6110, the IS6110 is inserted in the direct repeat locus in the repeat located between spacers 24 and 25. The predicted fragment size for this insertion in isolates with group 1 spoligotypes is 1.30–1.45 kb, depending on the number of spacers between spacers 25 and 36, where the PvuII site is located. The predicted fragment size for this insertion in isolates with group 2 or 3 spoligotypes is 4.51–4.58 kb, depending on the number of spacers between spacers 25 and 43 (the next PvuII site occurs outside of the direct repeat locus). Since the predicted fragment size for these 40 isolates was not consistent with the observed size, the insertion site in these isolates was sequenced. Sequencing showed that the isolates had a different insertion site (DK1) (19), which is very common among isolates with two copies of IS6110. The predicted fragment size for this insertion is 1.38 kb. This size is the one predicted for group 1 isolates and is a clear example of two isolates with different IS6110 insertions yielding PvuII fragments that are indistinguishable by the standard RFLP method.

## Summary

The TB genotyping network database demonstrates the diversity of strains that cause TB in the United States. The 10,883 patients in the study represented approximately 11.6% of all new cases of TB in the United States from 1996 through 2000. The sentinel sites were reasonably representative of the geographic and demographic diversity in the United States. Compiling this database from results submitted from seven laboratories was a considerable undertaking, and analyzing such a large collection of fingerprint patterns is difficult. From our quality assurance program and personal experience, we know that, even under the most carefully controlled conditions, IS6110 fingerprinting results are not 100% reproducible. We are certain that some of the fingerprint patterns, which were classified as different and received different designations, would have been identical had they been run side by side on the same gel. Also, as we have described, some fingerprint patterns for low-copy isolates appear identical but do not represent the same IS6110 insertions and thus do not represent closely related strains. Some of these difficulties resulted from the application of a rigid standard for defining distinct patterns, a process that is often subjective.

Even though some individual results may have been questionable, several clear conclusions emerged. Large sets of strains with related fingerprint patterns, not previously recognized, are spread across the United States. Given the rather slow rate of change in fingerprints, these must represent endemic strains that have circulated in the United States for decades. Consistent with this conclusion is the presence in the database of fingerprint patterns resembling the pattern of the laboratory strain H37 that was originally isolated in New York in 1905 (8). Many of the patterns in these sets represent single isolates, which suggests that they are the result of reactivation of remote infections acquired years or decades earlier. Analysis of the demographic characteristics of the patients will be required to confirm this observation. Among these large sets, outbreak strains (patterns) were generally restricted to a single sentinel site, as were clustered isolates in general.

We conclude that a large-scale, prospective comparison of fingerprint patterns from wide geographic regions is useful for research studies but is of limited value for TB control purposes. Comparisons of isolates from smaller areas are not only more meaningful but also more feasible. This limitation does not mean that searching multiple databases for specific fingerprint patterns, for example the “W” strain, is not useful in some circumstances.

The difficulties in analyzing IS6110 fingerprint patterns and the often slow turnaround time for obtaining results limit the value of this procedure to TB control programs. As an alternative, rapid, polymerase chain reaction–based testing, such as spoligotyping or mycobacterial interspersed repetitive units variable number of tandem repeats (MIRU-VNTR) analysis, would be a logical first step for universal genotyping of isolates. These methods provide greater reproducibility and give digital results, which simplify analysis. However, this approach has the following limitations. Many common spoligotypes were seen among low-copy-number isolates, although even IS6110 fingerprinting does not greatly improve resolution with these isolates. We also found that 9% of the isolates have W-Beijing fingerprint patterns that are known to have the same spoligotype; all isolates yielding the Beijing spoligotype would require IS6110 typing. Sufficient data are not available to predict the discriminatory power of MIRU-VTNR. However, preliminary results suggest that the combination of spoligotyping and VNTR typing will provide adequate resolution for most uses, thus limiting the need for additional typing by IS6110 fingerprinting.
